# Working Memory Deficits After Lesions Involving the Supplementary Motor Area

**DOI:** 10.3389/fpsyg.2018.00765

**Published:** 2018-05-23

**Authors:** Alba Cañas, Montserrat Juncadella, Ruth Lau, Andreu Gabarrós, Mireia Hernández

**Affiliations:** ^1^Department of Neurology, Hospital Universitari de Bellvitge L'Hospitalet de Llobregat, Spain; ^2^Department of Neurosurgery, Hospital Universitari de Bellvitge L'Hospitalet de Llobregat, Spain; ^3^Cognition and Brain Plasticity Group, Bellvitge Biomedical Research Institute (IDIBELL) L'Hospitalet de Llobregat, Spain; ^4^Section of Cognitive Processes, Department of Cognition, Development and Educational Psychology, University of Barcelona, Barcelona, Spain; ^5^Basque Center on Cognition, Brain and Language, Donostia, Spain

**Keywords:** executive control, neuropsychology, neurosurgery, processing speed, SMA syndrome, supplementary motor area, verbal fluency, working memory

## Abstract

The Supplementary Motor Area (SMA)—located in the superior and medial aspects of the superior frontal gyrus—is a preferential site of certain brain tumors and arteriovenous malformations, which often provoke the so-called SMA syndrome. The bulk of the literature studying this syndrome has focused on two of its most apparent symptoms: contralateral motor and speech deficits. Surprisingly, little attention has been given to working memory (WM) even though neuroimaging studies have implicated the SMA in this cognitive process. Given its relevance for higher-order functions, our main goal was to examine whether WM is compromised in SMA lesions. We also asked whether WM deficits might be reducible to processing speed (PS) difficulties. Given the connectivity of the SMA with prefrontal regions related to executive control (EC), as a secondary goal we examined whether SMA lesions also hampered EC. To this end, we tested 12 patients with lesions involving the left (i.e., the dominant) SMA. We also tested 12 healthy controls matched with patients for socio-demographic variables. To ensure that the results of this study can be easily transferred and implemented in clinical practice, we used widely-known clinical neuropsychological tests: WM and PS were measured with their respective Wechsler Adult Intelligence Scale indexes, and EC was tested with phonemic and semantic verbal fluency tasks. Non-parametric statistical methods revealed that patients showed deficits in the executive component of WM: they were able to sustain information temporarily but not to mentally manipulate this information. Such WM deficits were not subject to patients' marginal PS impairment. Patients also showed reduced phonemic fluency, which disappeared after controlling for the influence of WM. This observation suggests that SMA damage does not seem to affect cognitive processes engaged by verbal fluency other than WM. In conclusion, WM impairment needs to be considered as part of the SMA syndrome. These findings represent the first evidence about the cognitive consequences (other than language) of damage to the SMA. Further research is needed to establish a more specific profile of WM impairment in SMA patients and determine the consequences of SMA damage for other cognitive functions.

## Introduction

**Figure 1:**
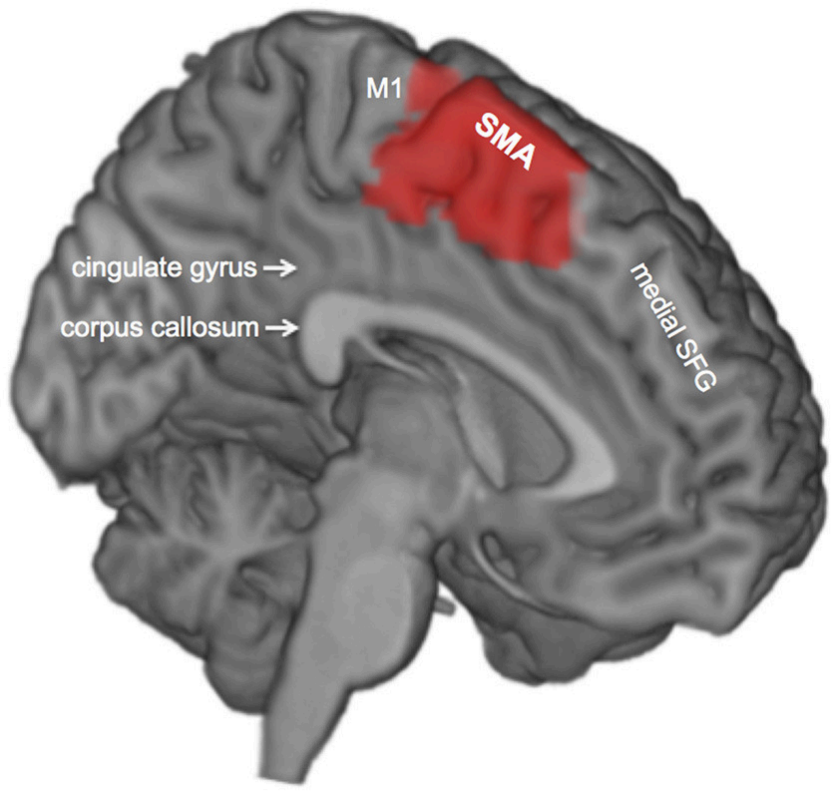
Medial view of the left hemisphere. The supplementary motor area is shown in red. SFG, superior frontal gyrus; SMA, supplementary motor area; M1, primary motor cortex.

The supplementary motor area (SMA) is situated in the superior and medial aspects of the superior frontal gyrus (Penfield and Welch, 1951), in front of the primary motor cortex (M1) and bordering inferiorly with the portion of the cingulate gyrus just above the genu of the corpus callosum (Talairach and Bancaud, 1966) (Figure 1). At a functional level, the SMA is fundamental in the selection, preparation, initiation, and execution of complex sequences of voluntary movements (Weilke et al., 2001). Such an important motor role relies on white-matter connectivity between the SMA and different core motor structures of the nervous system such as M1 (Catani et al., 2012), the striate body (Alexander and Crutcher, 1990; Lehéricy et al., 2004), or the spinal cord (Rizzolatti et al., 1996). The dominant SMA also plays a critical role in the control of motor aspects of speech production (Penfield and Rasmussen, 1950; Chassagnon et al., 2008), thanks to its connectivity with Broca's area (Vergani et al., 2014; Sierpowska et al., 2015).

The SMA happens to be a preferential site of different neurological disorders and abnormalities—most frequently tumors (especially low-grade gliomas; Duffau and Capelle, 2004) and epileptic foci (Chassagnon et al., 2008)—but cases with other etiology such as arteriovenous malformations (MAVs; Sailor et al., 2003) and cerebrovascular accidents (CVA; Dick et al., 1986; Ziegler et al., 1997; Pai, 1999) have also been described. This particularity has driven a special interest in investigating the functional symptoms derived from damage to the SMA, the collection of which is known as SMA syndrome (Laplane et al., 1977). The most apparent and common symptoms of the SMA syndrome consist of movement disorders, which are subject to the SMA somatotopy: the representation of the face, the contralateral upper limb, and the contralateral lower limb are located from middle to posterior SMA portions (Fontaine et al., 2002). Extreme cases show contralateral hemiparesis and hemiplegia, but patients most frequently show difficulties with fine hand movements, rapid alternating sequences, and bimanual coordination (Laplane et al., 1977; Rostomily et al., 1991; Zentner et al., 1996; Pai, 1999; Bannur and Rajshekhar, 2000). As the anterior portion of the SMA in the dominant hemisphere represents language (Fontaine et al., 2002), patients may also show language disorders if the lesion is located in the dominant SMA. These language disorders consist in varying degrees of transcortical motor aphasia (Masdeu et al., 1978; Alexander and Schmitt, 1980; Berthier et al., 1991; Ziegler et al., 1997): difficulties in the initiation of speech, reduced fluency (even mutism in extreme cases), relatively preserved repetition and reading skills, and comprehension difficulties with complex language content and speech at a high speed rate.

Many SMA lesions have surgical treatment, which results in several patients recovering the altered functions. Even so, 19.46% of patients show permanent motor and speech sequelae after surgery, often interfering with daily life (Gabarrós et al., 2011). In consequence, the bulk of SMA research has mainly focused on identifying what variables influence the likelihood that SMA surgical patients show permanent motor and speech disorders (Laplane et al., 1977; Rostomily et al., 1991; Zentner et al., 1996; Ziegler et al., 1997; Bannur and Rajshekhar, 2000; Duffau et al., 2001; Fontaine et al., 2002; Nelson et al., 2002; Peraud et al., 2002; Krainik et al., 2003, 2004; Russell and Kelly, 2003; Sailor et al., 2003; Hashiguchi et al., 2004; Liu et al., 2004; Yamane et al., 2004; Ulu et al., 2008; Rosenberg et al., 2010; Gabarrós et al., 2011; Anbar, 2012; Kim et al., 2013; Ryu et al., 2013; Nakajima et al., 2014, 2015; Satoer et al., 2014; Abel et al., 2015; Acioly et al., 2015; Ibe et al., 2016; Satter et al., 2017; Vassal et al., 2017). One of the most important findings in this respect has been that the probability of permanent motor deficits increases with the severity of such deficits before surgery (Gabarrós et al., 2011). Another important contribution of those studies has been the observation that awake mapping surgery significantly reduces motor sequelae (Gabarrós et al., 2011): it allows the exact identification of the SMA by having the patient conduct a motor task (generally, either a finger-opposition motor task or a bimanual coordination one) while using electrical stimulation that, when applied to the SMA, disrupts task performance. This exact identification helps avoid unnecessary damage to SMA's healthy portions during lesion resection and, in turn, better preserve the motor function. The identification of language areas of the SMA during awake mapping surgery has also been very effective in preserving speech (Duffau et al., 2003; Sierpowska et al., 2015): electric stimulation in areas relevant for language produces speech impairment during word-generation tasks, which allows mapping of those SMA areas to protect their language function during lesion resection. These findings have represented precious information for the development of SMA protocols. The refinement of such protocols is still a priority in clinical research, as they are critical to achieving the highest effectiveness in guiding the treatment plans of SMA lesions.

In contrast to the great amount of attention devoted to motor and speech disorders, the assessment of cognitive functions (other than language) has been neglected in SMA patients. Especially surprising is the lack of attention that has been given to working memory (WM): a cognitive mechanism whose attentional component (att-WM) allows sustaining information temporally so that its executive component (ex-WM) can manipulate such information and operate with it (Baddeley, 1992, 2003; Wingfield, 2016). In fact, many neuroimaging studies with both healthy and brain-damaged individuals have evidenced that the SMA is part of a widespread fronto-parietal network underlying WM [see Owen et al.'s (2005) and Rottschy et al.'s (2012) meta-analyses]. Most of those studies used classical WM tasks in cognitive research which especially challenge the ex-WM, such as the *n-back task*: the participant needs to indicate whether the current stimulus matches the one from *n* steps earlier in a continuous sequence, with the load factor *n* increasing to make the task progressively more difficult in terms of load. This is particularly relevant because ex-WM is essential for higher-order functions such as reasoning, problem solving, and learning (Engle et al., 1999; Shah and Miyake, 1999). This means that ex-WM deficits could compromise an individual's capacity to perform a wide range of complex cognitive tasks. Therefore, it is fundamental to determine whether SMA damage leads to WM impairment. A positive answer to this question would mean that WM deficits need to be considered as part of the SMA syndrome and, hence, taken into account in future refinements of clinical protocols.

Nakajima et al. (2014) conducted the only prior study investigating the effects of SMA damage on WM with the description of two patients with brain tumors situated in the SMA. Using a *2-back task* during awake mapping surgery, these authors obtained direct evidence that the SMA plays a role in WM, which is consistent with prior neuroimaging studies (Owen et al., 2005; Rottschy et al., 2012) that identified the SMA as one more region comprised in the WM fronto-parietal network. However, it is precisely because of the fact that WM relies on such a widespread network that it remains unclear whether permanent SMA damage would be relevant in patients' daily life. In other words, the rest of the network might overcome the permanent SMA dysfunction. Whether or not SMA damage has a real hampering effect on patients' WM can only be tested with data obtained outside the operating room. In this respect, Nakajima et al.'s (2014) data is uninformative because they merely reported the WM scores obtained by the two cases in the absence of a control group.

The main goal of the current study was to determine whether lesions involving the dominant SMA hamper WM. We focused on the dominant side because the SMA shows a certain degree of hemispheric dominance and, thus, more severe deficits are expected from damage in the dominant SMA compared to the non-dominant one (Rogers et al., 2004). With this main purpose, we conducted a group study with SMA patients and healthy controls.

We approached this study from an applied perspective using tasks from clinical practice, as opposed to experimental tasks typically used in basic cognitive research. In particular, we measured WM with the *WM Index* of the WAIS-III (Wechsler, 1997), which combines the scores of 3 tasks: *Digit span (Dspan), Letter-Number Sequencing (LNS)*, and *Arithmetic*. The *Dspan* tests the capacity of mentally maintaining a series of items (digits) in the same order they were memorized. Although the second part of this task also requires the mental manipulation of those items (i.e., retrieving them in a backward order), such manipulation is rather simple. Therefore, this task fundamentally measures att-WM. The *LNS* is much more demanding on ex-WM, as it requires a more complex mental manipulation of a series of items: numbers and letters are mixed and the participant is required to retrieve them in a specific order different from that in which they were memorized. *Arithmetic* is also particularly demanding on ex-WM, but the fact that the participant needs to mentally solve mathematical problems makes it much more complex than *LNS*.

We also included the *Processing Speed (PS) Index* of the WAIS-III in the testing to assess the possibility that WM deficits in SMA patients are reducible to PS difficulties (Fry and Hale, 1996): it has been observed that brain damage tends to reduce an individual's PS (see the WAIS-IV Technical and Interpretative Manual, Wechsler, 2008; see also Hawkins, 1998; Fisher et al., 2000). This is because WM holds information only briefly and, hence, the time to manipulate such information and perform mental operations with it is limited. In cases of remarkably slow PS, the first processed items may no longer be available when the last ones reach WM, making it impossible to perform any manipulation or operation with them.

As a secondary goal, we set out to examine whether cognitive deficits associated to SMA damage would go beyond WM and extend to another cognitive process that is also relevant for complex cognition: executive control (EC), understood as a set of cognitive processes that are needed for the flexible allocation of mental resources in the service of goal-directed behavior (Posner and Snyder, 1975; Miller, 2000; Badre, 2008; Kouneiher et al., 2009; Solomon et al., 2009). This additional question was based on the fact that the SMA is linked to the dorsolateral prefrontal cortex (DLPFC)—a relevant brain structure for EC—through white matter connectivity (Nachev et al., 2008). We addressed this question by comparing SMA patients and controls in the performance of a task that has been often used as a broad, quick EC measure in clinical contexts: verbal fluency (Shao et al., 2014). In particular, we used a *semantic* (animals) and a *phonemic* (letter P) verbal fluency: the participant is given a limited amount of time to retrieve all animals or words starting with the letter P as she can. In addressing this question, however, it is necessary to take into consideration that WM and EC processes are tightly related. For example, some EC processes, such as the ability to suppress interference from distracting stimuli, depend on an individual's WM capacity (Redick and Engle, 2006). Such an association has also been observed with the verbal fluency task. Indeed, verbal fluency performance has been associated with WM capacity in different studies, with both healthy individuals (Rosen and Engle, 1997; Rende et al., 2002; Azuma, 2004; Hedden et al., 2005; Unsworth et al., 2010) and neuropsychological patients (Sands et al., 2000; Witt et al., 2004; Lam et al., 2006; Larsson et al., 2008; Zahodne et al., 2008). Therefore, we controlled for by-product effects of WM when assessing the effects of SMA damage on verbal fluency.

## Material and methods

### Participants

**Figure 2:**
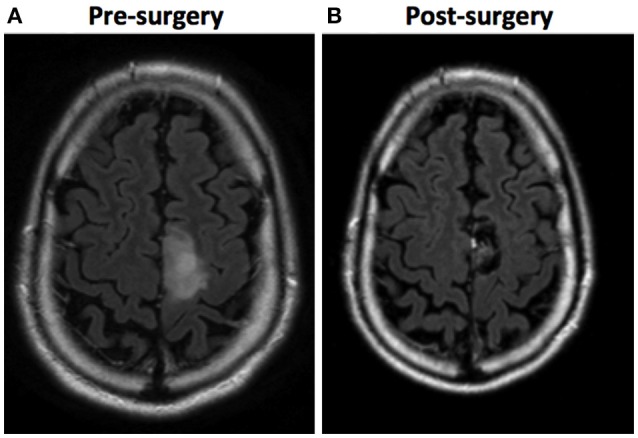
Example of an SMA lesion (in these images, left is right and right is left). **(A)** A FLAIR sequence MRI shows a tumoral lesion involving the left SMA in a 35-year-old patient. **(B)** A FLAIR sequence MRI 6 months after surgery shows a complete resection, achieved by performing awake brain mapping surgery. The pathology report was anaplastic oligodendroglioma.

Twenty-four participants took part in this study, half of whom were patients with a lesion involving the dominant SMA and the other half were control participants. The patient and control groups were matched for gender (7 men and 5 women per group), age (patients: mean = 41.25 years, *SD* = 10.9; controls: mean = 41.33 years, *SD* = 10.54) and years of educational attainment (patients: mean = 11.5, *SD* = 2.7; controls: mean = 12.33, *SD* = 2.67). This matching was conducted by pairing each patient with a control in all those three socio-demographic variables. All participants were right-handed. Patients were attended in the Neurosurgery Service of the *Hospital Universitari de Bellvitge* (HUB, Barcelona, Spain) between 2012 and 2015. The etiology of the lesion was a tumor between grade I and III in all cases except for a cerebral abscess and two AVMs. All lesions were located in the left cerebral hemisphere. All patients but one underwent lesion resection. As WM and EC were assessed with verbal tasks, patients diagnosed with language impairment by their clinical neuropsychologist were not included in the study. The absence of language impairment was determined by asking patients to describe the Cookie Theft Picture of the Boston Diagnostic Aphasia Examination (Goodglass and Kaplan, 1983), with which the neuropsychologist determined that the spontaneous speech of all patients was fluent, with information content, and free of anomia and phonological, semantic, morphological, or syntactic errors. Table 1 summarizes patients' socio-demographic characteristics and their type of SMA lesion (see also Figure 2 for an example of an SMA lesion before and after surgical treatment).

**Table 1:** Patients' socio-demographic and clinical information.

**Patient**	**Age**	**Gender**	**Educational attainment**	**SMA lesion**
p01	44	Female	High school	Abscess
p02	40	Female	High school	Tumor: astrocytoma
p03	58	Female	High school	Arteriovenous malformation
p04	30	Male	Middle school	Tumor: astrocytoma
p05	34	Male	Vocational training	Tumor: oligodendroglioma
p06	43	Male	Middle school	Tumor: anaplastic oligoastrocytoma
p07	56	Male	High school	Tumor: anaplastic oligoastrocytoma
p08	26	Male	Primary school	Arteriovenous malformation
p09	37	Female	Primary school	Tumor: anaplastic oligoastrocytoma
p10	33	Male	College	Tumor: oligodendroglioma
p11	58	Male	Primary school	Tumor: oligodendroglioma
p12	36	Female	College	Tumor: astrocytoma

*Educational attainment = the maximum educational level that the patient completed in the Spanish educational system*.

### Procedure

In the case of patients, experimental testing was included in the same clinical neuropsychology sessions, consisting in the assessment of language and fine hand movements. The testing session was conducted a few days before surgery. The experimental testing was repeated between 4 and 6 weeks after surgery. We addressed the main and the secondary questions in this study—the effects of SMA damage in WM and verbal fluency—using patients' pre-surgery data. We used patients' post-surgery data only to conduct complementary analyses examining post-surgery changes in WM, verbal fluency, and PS (see Data analyses and Results sections for further details).

### Tasks

#### WM assessment—WM index of the WAIS-III

The *WM Index* is derived from 3 tasks of the WAIS-III (Spanish Edition; Wechsler, 2001), which need to be administered in the same order as we describe them here: *Digit span (Dspan), Letter-Number sequencing (LNS)*, and *Arithmetic*. The sum of the raw scores for each task gives the raw score for the *WM Index*.

##### Dspan

This task is composed of two parts: the *Dspan forward* and the *Dspan backward*. For the *Dspan forward*, the experimenter utters strings of digits at a rate of one number a second, approximately. The participant needs to repeat those digits in the same order the examiner uttered them. There are 8 blocks with 2 trials for each string length, which starts with 2 digits and increases by 1 in each successive block. The administration of the task is terminated if the participant fails both trials within the same block. The same procedure is used for the *Dspan backward*, with the exception that the participant needs to repeat the digits in reverse order and that only seven (instead of eight) blocks compose the task. The *Dspan* raw score is the sum of the total number of correct *Dspan forward* and *Dspan backward* trials.

##### LNS

The experimenter utters strings of items consisting of different numbers and letters presented in mixed order and at a rate of one item a second, approximately. The participant needs to repeat each string, uttering first the numbers in numerical order followed by the letters in alphabetical order. There are 8 blocks with 3 trials for each string length, which starts with 3 items and increases by 1 in each successive block. The administration of the task is terminated if the participant fails all three trials within the same block. The *LNS* raw score is the sum of correct trials.

##### Arithmetic

The experimenter reads arithmetic problems out loud and asks the participant to give the correct answer to them. The participant is not allowed to take any written notes but the experimenter can repeat the entire problem once, if requested. Complexity in terms of the amount of information that needs to be held, the difficulty of the mathematical operations required (additions, percentages, etc.), and the time allotted for problem solving increases with every problem. There are 20 problems, and the administration of the task is terminated after the participant fails to solve 4 consecutive problems. The participant is given 1 point for each problem correctly solved, the total sum of which composes the *Arithmetic* raw score.

#### EF assessment—verbal fluency tasks

Verbal fluency tasks involve a limited amount of time (usually 1 min) for the participant to name as many words as possible according to a key, which is usually semantic or phonemic. We tested verbal fluency using both types of keys. On the *semantic fluency*, the participant had 1 min to generate as many words belonging to the semantic category “animals” as she could. On the *phonemic fluency*, the participant had the same amount of time to generate as many words as possible that began with the letter P, excluding proper names and repetitions of the same word with different endings. The raw score for each type of fluency (*semantic, phonemic*) is the total number of words generated within the allotted time limit.

#### PS assessment—PS index of the WAIS-III

The *PS Index* is derived from 2 tasks of the WAIS-III (Spanish Edition; Wechsler, 2001): *Coding-Digit Symbol (CDsymbol)*, and *Symbol Search (Ssearch)*. The sum of the raw scores for the 2 tasks gives the raw score for the *PS Index*.

##### CDsymbol

The participant is provided with a key matching each digit (from 1 to 7) with different meaningless symbols: e.g., digit 1 is associated with a horizontal double-headed arrow, digit 2 with a vertical double-headed arrow, digit 3 with 3 parallel horizontal lines, etc. Below this digit-symbol matching key—which remains always visible to the participant to avoid WM load effects—the participant is provided with a series of numbers, each placed above a blank box. She needs to draw the appropriate symbol in the box below each number according to the digit-association key. The participant is instructed to complete as many boxes as she can within the allotted time of 2 min. The *CDsymbol* raw score is the sum of the correctly completed boxes.

##### Ssearch

The participant is provided with 2 meaningless symbols in a left column. She needs to indicate if at least 1 of those 2 symbols is present in a group of 5 meaningless symbols in a right column. The participant gives the response by marking the “yes” or the “no” box. She is instructed to complete as many trials as possible in the allotted time of 2 min. The *Ssearch* raw score is the sum of correct answers minus the sum of errors.

## Data analyses

To reduce the skewness in the distribution of the data, we used transformed scores for data analyses. In the case of all WAIS-III tasks and verbal fluencies, we transformed raw scores into standard scores using Spanish normative data: WAIS-III Spanish edition (Wechsler, 2001) and NEURONORMA (Peña-Casanova et al., 2009), respectively. As for the *WM* and *PS Indexes*, we used intelligence quotient (IQ) scores: standard scores of the tasks composing each index were summed and transformed into IQs according to Spanish normative data (Wechsler, 2001). All data analyses were conducted with R (version 3.4.1; R Development Core Team, 2008).

Potential outliers were identified with box plots. In small samples it is difficult to determine whether an extreme observation is in fact outlying or merely reflects variability, especially in the case of patients. Therefore, we only considered as outlying the scores of control participants that fell below the cut-off for normality according to normative data. We based this action on the rationale that, by definition, controls cannot show impairment. This only occurred with two control participants: control c04 was not considered for any group analysis related to WM because he scored below the cut-off in most WM measures (Supplementary Figures 1A–C); control c06 was an outlier in *phonemic fluency* (Supplementary Figure 1D). In between-group analyses, we also excluded the data of the two patients matched socio-culturally with controls c04 and c06: p04's WM data, and p06's *phonemic fluency* data.

We first performed non-parametric between-group analyses (using the Mann Whitney U test) to examine whether the median (Mdn) of the patient group in the different tasks differed from that of the control group. Second, we assessed whether any potential impairment in WM observed in patients was actually driven by difficulties in PS. This assessment was done partialling out the influence of PS on WM measures in regression analyses and using the residuals resulting from those regressions to compare patients and controls. A similar procedure was used to assess whether between-group differences in verbal fluency were merely a by-product of patients' WM impairment rather than due to differences in EC. Finally, we included complementary analyses to examine whether the surgical treatment of the lesion caused any changes in WM, verbal fluency, or PS. To this end, we performed non-parametric within-group analyses (using the Wilcoxon signed rank test) to compare patients' pre- and post-surgery medians in the different cognitive measures.

## Results

Table 2 summarizes patients' and controls' scores in each WM, verbal fluency, and PS measure. It also summarizes the results of all between-group comparisons.

**Table 2:** Summary of participants' performance and between-group comparisons in all WM, PS, and verbal fluency measures.

	**WM Index**	**Dspan**	**LNS**	**Arithmetic**	**PS Index**	**CDsymbol**	**Ssearch**	**Phonemic fluency**	**Semantic fluency**
p01	73	5	6	7	75	7	4	5	3
p02	92	8	9	10	103	13	8	8	13
p03	90	10	9	7	81	7	6	7	6
p04	65^*^	4^*^	9^*^	7^*^	103	12	9	5	4
p05	83	10	6	7	103	10	11	2	7
p06	79	7	7	7	78	7	5	2^*^	3
p07	92	10	8	9	111	12	12	7	8
p08	96	11	10	8	98	11	8	8	7
p09	65	5	5	4	98	11	8	6	4
p10	120	13	12	16	114	12	13	15	16
p11	120	12	13	16	89	8	8	10	11
p12	116	13	12	14	117	14	12	12	11
**Patients' Mdn**	**92**	**10**	**9**	**8**	**100.7**	**8**	**11**	**7**	**7**
c01	110	11	12	13	103	11	10	9	8
c02	110	12	13	11	114	12	13	12	9
c03	110	12	13	11	109	11	12	11	7
c04	65^*^	5^*^	6^*^	3^*^	95	10	8	7	9
c05	114	12	16	10	103	9	12	10	11
c06	90	9	8	9	98	11	8	5	7
c07	120	12	14	15	103	12	9	13	14
c08	102	10	12	10	120	15	12	12	8
c09	100	7	12	12	111	12	12	10	7
c10	126	13	16	16	117	15	11	12	9
c11	132	15	17	15	106	12	10	11	13
c12	113	13	12	12	117	13	13	11	15
**Controls' Mdn**	**110**	**12**	**13**	**12**	**107.5**	**11.5**	**12**	**11**	**9**
W/*p*-value	92.5/0.038	84.5/0.118	105.5/0.003	89.5/0.059	104.5/0.062	105/0.056	95/0.186	95.5/0.022	98.5/0.13
W/*p*-value (residuals)	30.5/0.053	46/0.36	21.5/0.011	39/0.17				49.5/0.97	

*Numbers in participants' code indicate the patient-control pairing (e.g., c01 is the control participant for p01). WM and PS Indexes are measured in terms of intelligence quotient. The values for the rest of the measures are standard scores. W/p-value (residuals): the significance of the between-group comparison partialling out PS (WM measures) or WM (phonemic fluency)*.

* *Statistical values reported without considering these participants as they were considered outliers (or the patient pair of an outlier control participant)*.

### Between-group differences in WM

**Figure 3:**
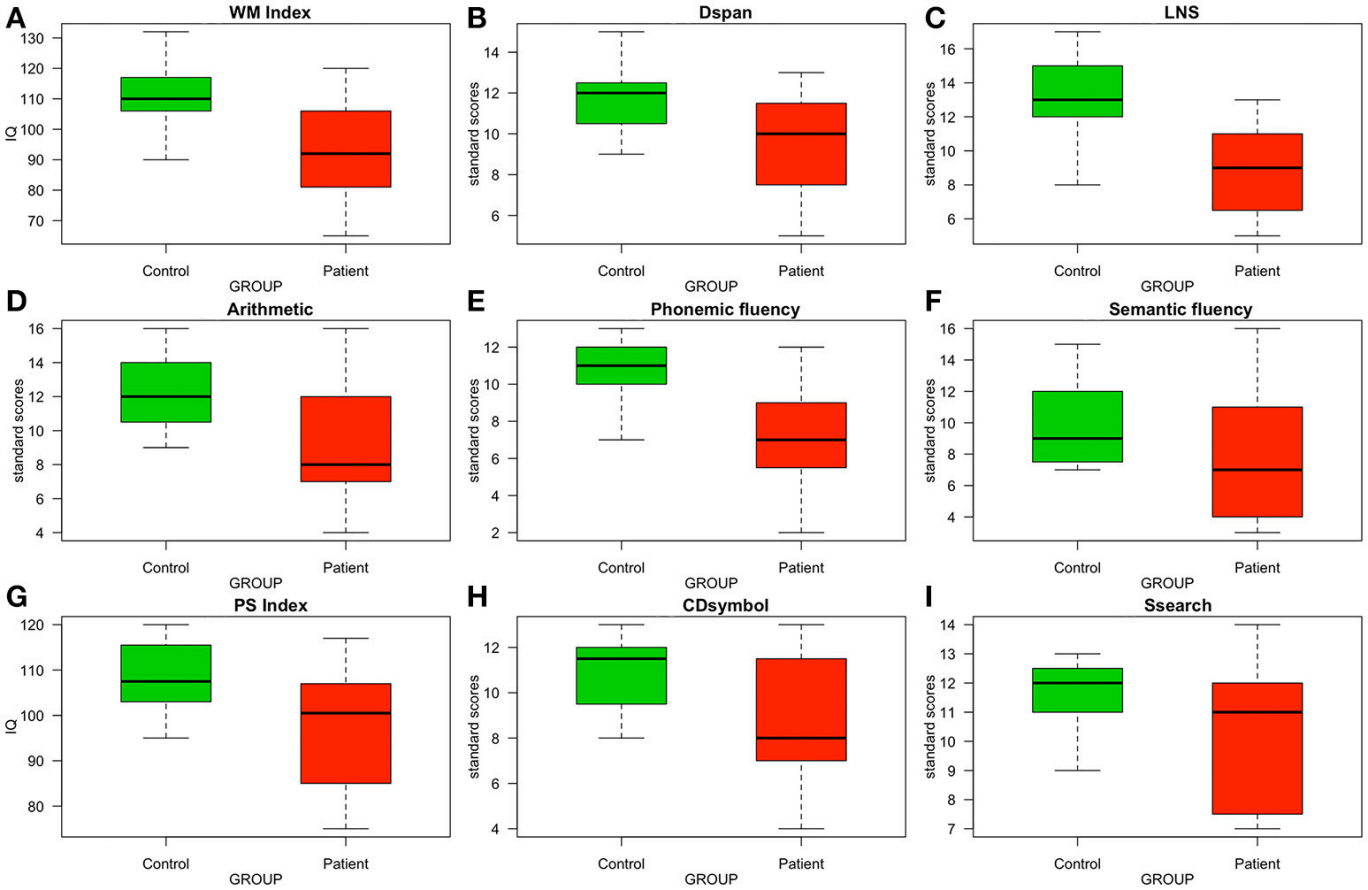
Boxplots comparing the control and the patient groups in measures of WM, EC, and PS. Patients' measures were taken in the pre-surgery phase. **(A–D)** WM measures. **(E,F)** EC measures. **(G–I)** PS measures. IQ, intelligence quotient.

The *WM Index* of patients (Mdn = 92) was significantly lower than that of controls (Mdn = 110; *W* = 92.5, *p* = 0.038), suggesting that SMA lesions led to WM impairment. To examine what WM task was more problematic for patients, we compared the performance of patients and controls in each WM task separately: patients clearly performed worse than controls on *LNS* (patients' Mdn = 9, controls' Mdn = 13, *W* = 105.5, *p* = 0.003); patients also showed poorer performance than controls in *Arithmetic*, being this between-group difference marginally significant (patients' Mdn = 8, controls' Mdn = 12, *W* = 89.5, *p* = 0.059); and there were no significant between-group differences in *Dspan* (patients' Mdn = 10, controls' Mdn = 12, *W* = 84.5, *p* = 0.118). These results suggest that, in general, patients performed worse than controls in WM tasks, with difficulties appearing in the ex-WM tasks (*LNS* and *Arithmetic*) rather than in the att-WM one (*Dspan*) (Figures 3A–D).

### Between-group differences in phonemic and semantic fluency

Patients performed poorly compared to controls in *phonemic fluency* (patients' Mdn = 7, controls' Mdn = 11, *W* = 95.5, *p* = 0.022), but not in *semantic fluency* (patients' Mdn = 7, controls' Mdn = 9, *W* = 98.5, *p* = 0.13). That is, patients showed reduced verbal fluency if tested with the *phonemic fluency* task (Figures 3E,F).

### Between-group differences in PS

Figure 3G shows a marginally reduced *PS Index* for patients compared to controls (patients' Mdn = 100.5, controls' Mdn = 107.5, *W* = 104.5, *p* = 0.062). Such reduction in PS was mainly driven by patients performing worse than controls in *CDsymbol* (Figure 3H; patients' Mdn = 8, controls' Mdn = 11.5, *W* = 105, *p* = 0.056) rather than in *Ssearch* (Figure 3I; patients' Mdn = 11, controls' Mdn = 12, *W* = 95, *p* = 0.186).

### PS influence on WM

Given the marginal impairment in PS shown by patients, we examined whether patients' difficulties with WM could be merely driven by slow PS. To this end, we first partialled out the contribution that PS had on participants' WM performance with linear regression analyses. We conducted a separate regression—including both controls and patients—for each WM measure (dependent variables). We used *CDsymbol* performance as the predictor factor, which was the PS task in which patients and controls differed the most. Then, we compared the residuals of controls and patients resulting from those regressions: significant between-group differences in these residuals would mean that patients and controls differed in WM regardless of PS. The results of these analyses showed a reduced *WM Inde*x for patients compared to controls (*W* = 30.5, *p* = 0.05), which was mainly driven by a poorer performance in *LNS* (*W* = 21.5, *p* = 0.011) rather than *Dspan* (*W* = 46, *p* = 0.36) or *Arithmetic* (*W* = 39, *p* = 0.17). *Arithmetic* seemed to be the WM task most influenced by PS, as the marginal between-group difference reported above vanished after controlling for PS. In spite of that, after removing the influence of PS on WM, patients showed a similar pattern of WM difficulties as the one reported above: they still showed a reduced *WM Index* due to poor performance on an ex-WM task: *LNS*. This indicates that patients' difficulties in ex-WM were genuine and not reducible to slow PS.

### WM influence on phonemic fluency

As exposed in the Introduction, besides EC processes, verbal fluency tasks also engage WM. This means that patients' reduced verbal fluency observed in the *phonemic* task version could have been driven by difficulties with its WM component rather than by EC deficits. To examine this possibility, we first partialled out the contribution of WM on participants' performance in *phonemic fluency* (dependent variable) with a linear regression analysis that included both controls and patients. We used *LNS* performance as the predictor factor in this regression, which was the WM task in which patients showed difficulties. Then, we compared controls and patients in the resulting residuals: significant between-group differences in these residuals would mean that patients and controls differed in the EC processes typically engaged by verbal fluency tasks regardless of their WM component. Contrasting with this prediction, however, the results showed no between-group differences in the residuals (*W* = 49.5, *p* = 0.97), indicating that patients' had no difficulties in engaging the EC processes required by the task. In other words, the reduced *phonemic fluency* reported above seemed to have been driven by patients' WM impairment.

### Complementary analyses on post-surgery changes

Prior studies focused on movement and language seemed to indicate that the surgical treatment of the lesion reduces the likelihood that patients remain with permanent sequelae (Gabarrós et al., 2011). In this study, however, resecting the SMA lesion did not result in any changes with respect to WM or PS function, whereas patients' performance in verbal fluency tended to worsen: *WM Index* (pre-surgery Mdn = 90, post-surgery Mdn = 86, *V* = 21, *p* = 0.759), *Dspan* (pre-surgery Mdn = 10, post-surgery Mdn = 8, *V* = 16, *p* = 0.733), *LNS* (pre-surgery Mdn = 9, post-surgery Mdn = 8, *V* = 21, *p* = 0.268), *Arithmetic* (pre-surgery Mdn = 7, post-surgery Mdn = 9, *V* = 21, *p* = 0.67), *PS Index* (pre-surgery Mdn = 103, post-surgery Mdn = 92, *V* = 45, *p* = 0.306), *CDsymbol* (pre-surgery Mdn = 8, post-surgery Mdn = 9, *V* = 26, *p* = 0.719), *Ssearch* (pre-surgery Mdn = 11, post-surgery Mdn = 10, *V* = 43, *p* = 0.125), *phonemic fluency* (pre-surgery Mdn = 7, post-surgery Mdn = 4, *V* = 45.5, *p* = 0.072), and *semantic fluency* (pre-surgery Mdn = 7, post-surgery Mdn = 5, *V* = 38, *p* = 0.074). Many different factors may play a role in the post-surgery outcome of cognitive processes, including potential collateral effects of surgery, premorbid cognitive deficits, and various clinical variables such as extension and exact location of the lesion. Since we did not control for many of those factors, we will not discuss the post-surgery outcome any further.

## Discussion

The main goal of the present study was to determine whether lesions affecting the SMA hamper WM by taking into consideration potential PS confounds. As a secondary goal we asked whether SMA damage would also hamper patients' performance on a broad, quick EC measure such as verbal fluency. We addressed these questions using tests commonly used in clinical practice to facilitate the transfer of the results to a health care context.

### On the impact of SMA lesions on WM

The results indicated that SMA lesions indeed compromised WM and that such a deficit could not be reduced to PS difficulties even though patients showed a trend toward slower PS compared to controls. The awake surgery data reported in Nakajima et al. (2014) had already evidenced a relation between SMA and WM. Neuroimaging data had also evidenced such relation. Indeed, different meta-analyses have attributed to the SMA a role in WM, regardless of the specific WM task, modality (verbal vs. non-verbal), and difficulty (Owen et al., 2005; Rottschy et al., 2012). However, neuroimaging data had also revealed that WM relies on a widespread fronto-parietal network, being the SMA one more of the various regions comprising such network along with portions of dorsolateral prefrontal, ventrolateral prefrontal, parietal, and subcortical regions (Owen et al., 2005; Rottschy et al., 2012). It is worth noting that such an extensive network could alleviate the effects of SMA damage to the extent that such damage would not have a significant impact on WM functionality. A neuropsychological approach was fundamental in shedding light onto this issue. However, WM deficits associated with SMA damage have never been previously reported, at least to our knowledge: as exposed in the Introduction, Nakajima et al.'s (2014) was the only study exploring the effects of SMA damage on WM, but the data obtained outside the operating room for the two single cases they reported were inconclusive. Therefore, the results of the present study represent the first evidence that the role of the SMA is relevant enough as to significantly impair WM in case of damage. In this regard, it is worth noting that, rather than the SMA playing a direct role in WM, it may play an indirect role through its connectivity with the rest of the brain regions composing the fronto-parietal network. This is a question for further research.

As for the specific pattern of WM difficulties, patients showed a reduced *WM Index*, which is a global WM measure. Among the different WM measures we had, WM deficits were captured by the *LNS* task. Patients did not differ from controls in *Dspan*, and *Arithmetic*, especially after removing the effects of PS. The fact that patients performed similarly to controls in *Dspan* indicated that they were relatively able to hold a series of items in WM. This means that their poor performance in manipulating such items (ex-WM)—which is what *LNS* mainly measures—could not be explained by patients' not having those items available. One would expect that this difficulty in manipulating information held in WM would, in turn, lead patients to perform poorly in tasks requiring operating with such information, as is the case with *Arithmetic*. It is relevant to note, however, that *Arithmetic* may not be the most valid tool to assess WM, as it depends on individuals' mathematical skills (Hill et al., 2010). In fact, in a study examining which tasks of the WAIS-III were the best predictors of WM capacity, Hill et al. (2010) found that *Arithmetic* was not among those predictors. These authors used common WM paradigms in experimental psychology as the gold standard in a study with 188 healthy individuals. The results of regression analyses indicated that, when considering only the 3 tasks originally thought to compose the *WM Index* (*Dpan, LNS*, and *Arithmetic*), *Dpan* and *LNS* accounted for most part of the variances (33% and 28%, respectively). In contrast, the *Arithmetic* was excluded from the model, evidencing its weak association with WM.

It is also relevant to point out that the present results should be taken only as a starting point for further research in which more sophisticated WM tools would be used. We acknowledge that the information one can gather with the *WM Ind*ex of the WAIS-III regarding individuals' WM functioning is limited. Future research with experimental tasks is needed to fully understand the profile of WM difficulties associated to SMA damage. A related question for future research is whether such profile is specific to SMA patients with respect to the WM profile of patients with damage in other regions within the fronto-parietal network.

### On the effects of SMA lesions on verbal fluency

The secondary question of this study was to determine whether cognitive impairment (other than language) associated to SMA goes beyond WM and also affects EC due to its connectivity with the DLPFC (Nachev et al., 2008). To this end, we compared patients' and controls' performance in verbal fluency, both *semantic* and *phonemic*. In first between-group analyses, patients showed worse performance than controls in the *phonemic* task version. Subsequent analyses, however, revealed that the WM processes engaged by the *phonemic fluency* task drove such a disadvantage. That is, it seems that patients' reduced *phonemic fluency* was most likely not due to difficulties in the EC processes required by this task but to the WM ones. Note, however, that it would be too precipitate to state that these results suggest that SMA damage does not lead to EC deficits. This is because verbal fluency is too broad of an EC measure. In addition, it is not possible to disentangle the multiple EC processes this task may engage (e.g., inhibition, response suppression, and switching, among others; Abwender et al., 2001; Hirshorn and Thompson-Schill, 2006), which is a relevant limitation because patients may have selective difficulties in some of these processes. An additional limitation of verbal fluency is its strong dependence on the integrity of other cognitive processes different from EC, such as lexical access or semantic processing (Shao et al., 2014). Therefore, our data only indicates that SMA damage does not seem to affect cognitive processes engaged by verbal fluency other than WM. Future research using more extensive and specific experimental protocols is needed to further investigate the potential association of EC dysfunction and SMA damage.

A secondary point related to the verbal fluency task is the fact that patients did not differ from controls in the *semantic* task version (before partialling out the effects of WM). This observation suggests that *semantic fluency* engaged WM processes to a lesser extent than *phonemic fluency* and, thus, was not affected by the collateral effect of patients' WM impairment. In fact, in some authors' view, *semantic fluency* is particularly associated with lexical-semantic skills rather than WM or EC (Shao et al., 2014). In line with this view, neuropsychological studies have found that patients with brain damage that compromises regions typically associated with lexical-semantic representations show more difficulties with the *semantic* than with the *phonemic* verbal fluency task (Jones et al., 2006; Laws et al., 2010; Magaud et al., 2010; Meijer et al., 2011). Similarly, Satoer et al. (2014) described a patient with the SMA syndrome whose reduced *semantic* (but not *phonemic*) verbal fluency seemed to stem from his language difficulties rather than his WM or EC dysfunctions.

### On SMA patients' slow PS

As exposed above, patients showed a marginal PS impairment, which did not drive their WM impairment. Beyond this fact, it is worth discussing the potential origin of such marginal PS difficulties. In this respect, it is relevant to point out that there is no specific brain region subserving PS, but it depends on white-matter pathways that synchronize the transmission of information across distributed brain networks (Mesulam, 1998, 2000). This is why the neurological conditions most commonly affecting PS are those involving axonal damage, such as multiple sclerosis or traumatic brain injury (Rao, 1996; Levine et al., 2006). In a neuroimaging study using the *CDsymbol* task as a PS measure, Turken et al. (2008) revealed that, among the various white-matter pathways critical for PS, at least two of them involve the frontal cortex: the superior longitudinal fasciculus (a major fronto-parietal tract) and fronto-striatal projections. One of the frontal locations most relevant seemed to be the superior frontal cortex (SFC) to which parietal regions project through the superior longitudinal fasciculus. Another relevant frontal site was the DLPFC, connected with the basal ganglia through fronto-striatal projections. It is of note that the functioning of both frontal structures might be—at least to a certain extent—compromised by SMA lesions: the SMA is, in fact, harbored in the superior and medial aspects of the SFC (Penfield and Welch, 1951) and linked to the DLPFC though white matter connectivity (Nachev et al., 2008). Therefore, the marginal PS impairment may have been due to the SFC and DLPFC (presumed) dysfunction having an impact on their parietal and striatal white matter links, respectively.

### Limitations of the current study

As acknowledged above, one of the limitations of the present study has to do with the simplicity of the tasks used to index WM and EC. In addition, the modest sample size has been a limitation in our statistical analyses. In this respect, it is worth underlining that the SMA's susceptibility to harbor tumors and other abnormalities calls for research on the SMA syndrome. However, the incidence of SMA damage is not massive, making it difficult to compose a patient group of a decent size: for example, the 12 patients in this study were recruited over a 3-year period in a hospital of reference for the treatment of SMA lesions in Spain. In fact, large samples (*n* > 15) are exceptional (Zentner et al., 1996; Peraud et al., 2002; Duffau et al., 2003; Russell and Kelly, 2003; Rosenberg et al., 2010; Kim et al., 2013), whereas single-case descriptions have been very common (Laplane et al., 1977; Masdeu et al., 1978; Dick et al., 1986; Ziegler et al., 1997; Pai, 1999; Duffau et al., 2001; Chung et al., 2004; Hashiguchi et al., 2004; Iwasaki et al., 2009; Ryu et al., 2013; Nakajima et al., 2014; Acioly et al., 2015; Satter et al., 2017). Some studies have been able to describe series of 6 and 7 patients (Rostomily et al., 1991; Bannur and Rajshekhar, 2000; Sailor et al., 2003; Liu et al., 2004; Yamane et al., 2004; Tankus et al., 2009; Vassal et al., 2017). In several other studies—including the present one—the series of patients has been large enough (8–15 patients) as to allow non-parametrical group analyses (Lim et al., 1994; Fontaine et al., 2002; Nelson et al., 2002; Krainik et al., 2004; Ulu et al., 2008; Gabarrós et al., 2011; Anbar, 2012; Abel et al., 2015; Nakajima et al., 2015; Ibe et al., 2016). In this regard, it is worth highlighting that this is the first group study investigating the effects of SMA lesions on cognitive functions other than language: the only prior study assessing non-linguistic cognitive functions only provided descriptive data of 2 single cases (Nakajima et al., 2014). In this respect, the results of the present study represent a remarkable new contribution to the research field. Even so, we interpret such results with caution.

It is also worth mentioning that a number of clinically related variables—which we did not control for—may have an influence on the extent of functional deficits. Two of these variables are the size and exact location of the lesion. In this respect, distinguishing between the 2 anatomical portions of the SMA—the pre-SMA, and the SMA-proper—would be of particular relevance. It seems that the former could be more related to cognitive processes (Chouinard and Paus, 2010). Therefore, it is possible that WM deficits arise only if the lesion affects the pre-SMA. In the case of a tumor, whether it invaded or displaced the SMA may also make a difference. In addition, it has been reported that the damage a tumor may cause is much greater if it is infiltrating (Gabarrós et al., 2011). Similarly, the degree to which (if any) the contralateral non-dominant SMA takes over the functions of the dominant one may vary across patients. It is worth noting, however, that a considerable large sample of patients would be needed to apply sound methodological approaches (e.g., neuroimaging studies based on lesion mapping techniques) and sound statistical methods (e.g., mixed-model regression analyses) in order to examine the implications of these variables.

## Conclusion

The results of this study represent the first evidence that WM impairment is a symptom of the SMA syndrome, which seems to stem from difficulties in manipulating information held in WM. PS is also somewhat compromised in SMA patients. However, WM deficits are not reducible to PS difficulties. These findings highlight the need to include WM assessment in clinical SMA protocols. Further research is needed to establish the specific WM profile of SMA patients and determine the consequences of SMA damage for other cognitive functions such as EC.

## Ethics statement

This study was reviewed and approved by the ethics committee of the *Hospital Universitari de Bellvitge* (L'Hospitalet de Llobregat, Barcelona, Spain). The study was conducted in accordance with the Declaration of Helsinki. All participants provided written informed consent.

## Author contributions

All authors listed, have made substantial, direct and intellectual contribution to the work, and approved it for publication.

### Conflict of interest statement

The authors declare that the research was conducted in the absence of any commercial or financial relationships that could be construed as a potential conflict of interest.

## Supplementary material

Supplementary Figure 1 Boxplots showing outlier control subjects in different WM and EC measures. **(A–C)** WM measures in which control c04 was an outlier. **(D)** EC measure in which control c06 was an outlier.
