# Supplementary figures and images for: Working Memory Deficits After Lesions Involving the Supplementary Motor Area
Source: Front Psychol. 2018 May 23;9:765. doi: 10.3389/fpsyg.2018.00765 (PMC5974158; doi:10.3389/fpsyg.2018.00765)

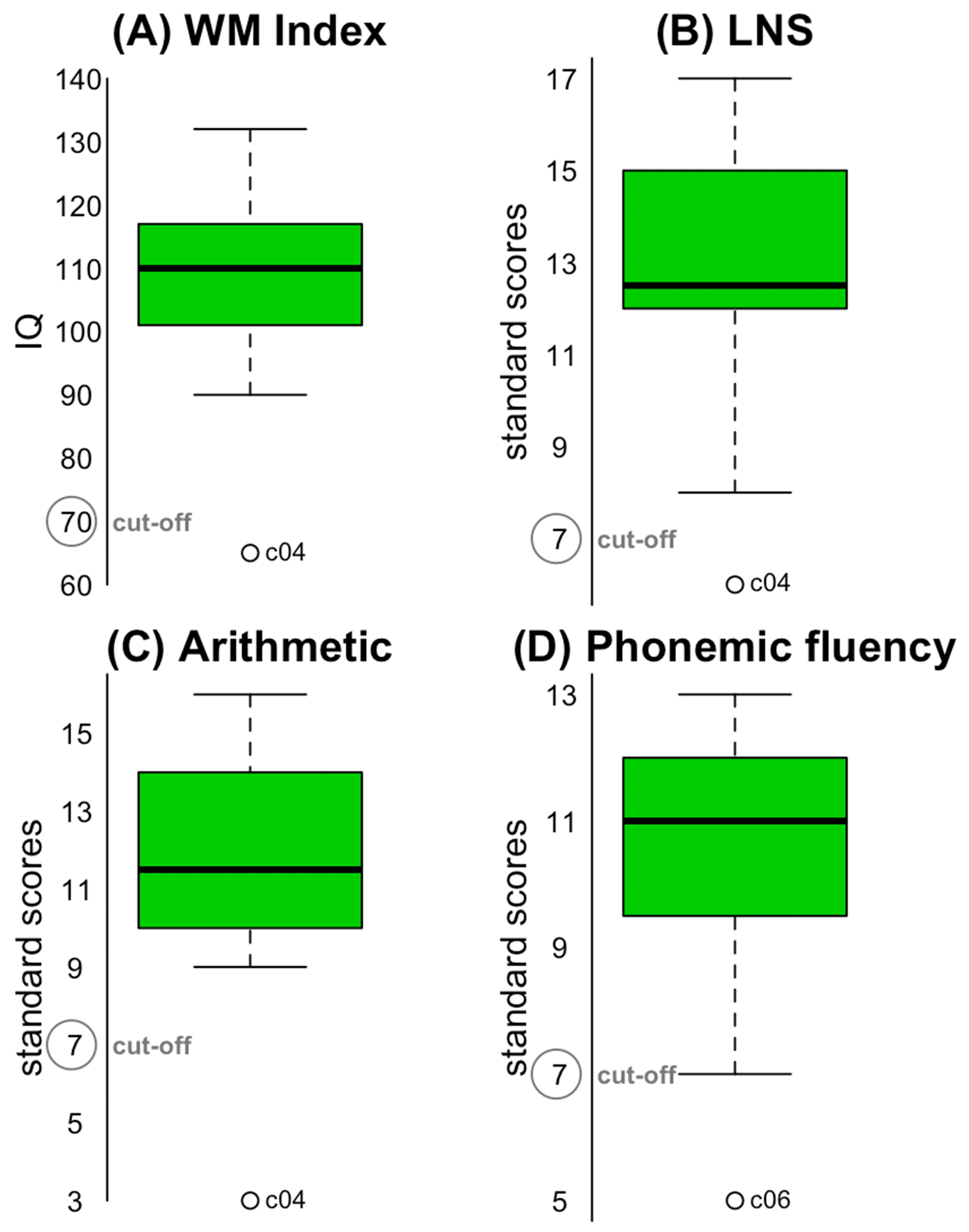

Supplement: Supplementary Figure 1 — Boxplots showing outlier control subjects in different WM and EC measures. (A–C) WM measures in which control c04 was an outlier. (D) EC measure in which control c06 was an outlier. [file Image1.TIF]
